# Agent-Oriented Goal Models in Developing Information Systems Supporting Physical Activity Among Adolescents: Literature Review and Expert Interviews

**DOI:** 10.2196/24810

**Published:** 2021-05-19

**Authors:** Kerli Mooses, Kuldar Taveter

**Affiliations:** 1 Institute of Computer Science University of Tartu Tartu Estonia

**Keywords:** agent-oriented goal models, physical activity, adolescent

## Abstract

**Background:**

Information and communication technologies (ICTs) are becoming increasingly popular in supporting the fight against low physical activity (PA) levels among adolescents. However, several ICT solutions lack evidence-based content. Therefore, there is a need to identify important features that have the potential to efficiently and consistently support the PA of adolescents using ICT solutions.

**Objective:**

This study aims to create evidence-based models of requirements for ICT solutions supporting PA by combining scientific evidence from literature and health experts. In addition, we test the suitability of agent-oriented goal models in this type of modeling process.

**Methods:**

A literature search of PubMed, Web of Science, and Scopus databases was conducted to identify evidence-based functional, quality, and emotional goals that have previously been proven to be relevant in supporting PAs among youth using ICT solutions. The identified goals were presented in the form of goal models. These models were used to collaborate with health experts to receive their input on the topic and suggestions for improvement. The initial goal models were improved based on the feedback from the experts.

**Results:**

The results indicated that agent-oriented goal modeling is a suitable method for merging information from the literature and experts. One strength of agent-oriented goal models is that they present emotional requirements together with quality and functional requirements. Another strength is the possibility of presenting results from a literature review in a systematic manner and using them thereafter in the communication process with stakeholders. Agent-oriented goal models that were created were easy to understand for health experts without previous experience in requirements engineering, which facilitates and supports collaboration with nontechnical stakeholders.

**Conclusions:**

The proposed agent-oriented goal models effectively merged information from scientific literature and experts in the field and presented early functional, quality, and emotional requirements in a holistic and coherent manner. We believe that the created models have high potential to help requirements engineers and developers to provide more efficient ICT solutions that support PA among adolescents in the future.

## Introduction

### Background

Despite the numerous health benefits of physical activity (PA) on mental, social, and physical health [1-3], countries struggle with low levels of PA among children and adolescents [4]. According to data from 32 European and North American countries, only 23% of adolescent boys and 14% of girls meet the recommended daily PA levels [4], which is a minimum of 60 minutes of moderate- to vigorous-intensity PA every day [5]. According to the studies, PA declines during adolescence [6,7]. At the same time, many health-related behavior habits are established during adolescence that carry on to adulthood [8,9]. Therefore, adolescence is a sensitive period that can influence health behavior in later life, and greater attention should be paid to supporting the PA of adolescents [9].

Numerous interventions have been developed to support PA among children and adolescents, with most of them being conducted in a school setting [10], where it is easy to reach all children despite their age or socioeconomic background. In recent years, interventions using a variety of technological solutions have emerged, which can be explained by the high level of technology use among children and adolescents as well as by the vast possibilities of information and communication technologies (ICTs) that enable more individualized behavior change interventions [10]. It is also important that through ICT solutions, it is possible to provide health information to people who would otherwise not have access to health education resources [11]. According to adolescents themselves, supporting PA by using technology makes PA more appealing and helps to increase their awareness of the actual activity levels [12]. Previous ICT interventions have shown promising results [10,13-20], irrespective of the technological mechanisms used to deliver or support the delivery of an intervention. Some examples of the mechanisms used are the internet [14,15,21], text messaging, email [19-21], and phone apps [22]. However, the development process of ICT solutions supporting PA includes several challenges, which is highlighted by the fact that not all ICT interventions have been proven to be effective or successful [22,23]. It has been pointed out that a stronger collaboration between scientists and technologists who develop the solutions is needed [22,24,25], as ICT interventions are more likely to be successful when combined with theory, such as behavior change theory [15,21]. Collaboration with health experts and scientists during the problem identification and requirements engineering phase helps to elicit evidence-based functional, quality, and emotional requirements for ICT-based intervention solutions that have the potential to support the behavioral change of the target group. Such collaboration with scientists and experts would also increase the quality of information, and according to a review, several apps lack evidence-based content and focus mainly on functionality, aesthetics, and engagement [22].

In addition to functional requirements, the quality and emotional side of the software application must also be considered because the adaptation of the application depends on the emotions of the user [26]. The lack of user-friendliness and being unappealing to the target group has been marked as a significant cause of dropout and low levels of involvement in previous interventions [23,27]. Therefore, it is crucial that the development process includes both end users [27] as well as different stakeholders, health experts, and scientists, which ensures that the end product will have an expected impact on the end users [25,28-30]. The method used to involve different stakeholders must be easy to comprehend, as stakeholders often lack previous experience in requirements engineering. At the same time, it should capture as many functional, quality, and emotional requirements that constitute an important input for requirements engineers and developers.

### Agent-Oriented Goal Modeling

One method for presenting emotional requirements for sociotechnical systems (STSs) is agent-oriented goal modeling [29-32]. STS is a software-intensive system that has defined operational processes followed by human operators and operates within an organization and comprises both social and technical aspects [33]. The STS consists of humans, software, and hardware [34]. According to the preceding definitions, ICT solutions supporting PA among adolescents fit into the category of STSs. Agent-oriented goal modeling [31,32,35] is a state-of-the-art method for eliciting and representing the requirements of STSs. In this method, the starting point is something to be achieved or done, whereas it is not yet important *who* or *what* does it. A distinct feature of motivational modeling is its explicit support for eliciting and representing emotional requirements [30,36]. The method enables the representation of functional, quality (nonfunctional), and emotional requirements for STSs in a holistic manner as a hierarchical goal tree, consisting of functional, quality, and emotional goals [30-32,35,36]. Agent-oriented goal modeling has been applied previously in several studies, which have shown that this approach is suitable and comfortable for including both technical and nontechnical stakeholders [30,31,36-38]. Moreover, this approach supports communication with nontechnical experts and other stakeholders whose input is required for the design process. Agent-oriented goal models provide a thorough description of possible solutions for the problem in question. The created models are not final technical requirements but rather offer an overall picture of the necessary elements and features that should be considered during the development process. On the basis of the agent-oriented goal models, requirements engineers can form more concrete requirements in the form of, for example, user stories [39], which can then be used in the further development process. Often, the creation of goal models includes a workshop or focus group with stakeholders to identify functional, quality, and emotional goals and the roles required for the attainment of the goals [30-32,36], which is followed by creating goal models based on the information gathered. However, in the current situation caused by the spread of COVID-19, the possibilities for face-to-face workshops have become limited, and alternative solutions have to be considered. In addition to expert opinions, more scientific evidence is emerging with regard to the features and factors that increase the effectiveness of ICT solutions supporting PA, which should also be considered in goal models.

### Objective

The main aim of our study is to create evidence-based models for ICT solutions supporting PA by combining scientific evidence from the literature and opinions of health experts. For this purpose, we also aim to pilot the use of agent-oriented goal modeling in the process of communicating the results of the literature review and involving experts with nontechnical backgrounds. Interestingly, we have previously used a similar approach in problem domains where arranging co-design workshops would be complicated, impossible, or even dangerous [37,38].

## Methods

### Overview

Previous studies using agent-oriented goal models have often gathered their input for goal models only from stakeholders through interviews or workshops [30,32,35,36]. For the topic under discussion, there is some scientific evidence on the features that have the potential to increase the effectiveness of ICT solutions supporting PA among adolescents and reduce dropout. Therefore, to combine the findings from previous ICT intervention studies with information from experts, this study was designed in the following steps:

- Identification of evidence-based features of ICT solutions that have the potential to support the behavioral change and PA of adolescents based on a literature review
- Development of initial goal models
- Inclusion of experts, model validation, and improvement

### Literature Review

First, we identified from the scientific literature evidence-based features of ICT solutions that have the potential to support the behavioral change and PA of adolescents. For this purpose, we searched for articles in the PubMed, Web of Science, and Scopus databases. Separate searches for three topics were conducted for each database. The topics and keywords used are listed in Textbox 1. The first search focused on reviews that covered ICT-based interventions with adolescents. The results of the first search indicated that behavior change techniques (BCTs) have the potential to positively influence the behavior of the target audience [15,21]. Considering this, the following search focused on identifying the most prevalent and effective BCTs used in ICT solutions aimed at adolescents. To gain more thorough insight into the needs and preferences of adolescents concerning ICT solutions and PA, the third search focused on qualitative studies. The reference list of each identified article was also searched for relevant reviews and studies.

The reviews that addressed ICT-based interventions with adolescents included different study methodologies, such as randomized controlled trials, quasi-experimental studies, and feasibility studies [14,15,17-21,24,40-43], providing a good overview of the features that have the potential to support PA levels. Information about BCTs was mostly elicited from studies or reviews describing ICT solutions using BCTs [22,25,44-46]. Finally, qualitative studies helped to identify possible facilitators and barriers perceived by the adolescents themselves [7,12,23,47-58].

**Search topics and keywords.**

**Search: reviews covering information and communication technology–based interventions with adolescents**

- “(adolesc*[Title/Abstract]) AND (review[Title/Abstract]) AND (‘physical activity’[Title/Abstract]) AND ((technology[Title/Abstract]) OR (web[Title/Abstract]) OR (E-Health[Title/Abstract]) OR (eHealth[Title/Abstract]) OR (mHealth[Title/Abstract]) OR (app*[Title/Abstract]))”

**Search: behavior change techniques used in information and communication technology solutions aimed at adolescents**

- “((adolesc*[Title/Abstract]) OR (young [Title/Abstract])) AND (‘physical activity’[Title/Abstract]) AND (‘behaviour change’[Title/Abstract])”

**Search: qualitative studies to identify factors valued by adolescent users**

- “(‘physical activity’[Title/Abstract]) AND ((qualitative[Title/Abstract]) OR (‘focus group’[Title/Abstract])) AND ((app*[Title/Abstract]) OR (mHealth[Title/Abstract]) OR (eHealth[Title/Abstract])) AND (adolesc*[Title/Abstract])”

### Development of Initial Goal Models

**Figure 1:**
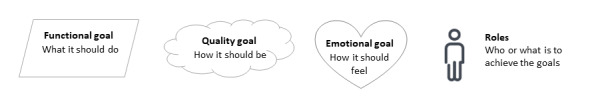
Notation for goal modeling.

The data gathered from the literature were used to create the initial version of goal models, which included three types of goals: *do, be,* and *feel* goals. *Do* goal is a functional goal that indicates what the system should do. *Be* goals or quality goals present the nonfunctional requirements of the system, which describe the quality aspects of the system—how the system should be. *Feel* goals are emotional goals that describe how the user should feel when interacting with the system. In addition, *roles* are identified, which represent who is responsible for the attainment of which goals. This information is eventually presented as a simple graph in which the functional goals are rendered in a tree-like hierarchy. In the hierarchical goal model, each subgoal represents a particular aspect of achieving its parent goal. The functional goals are represented with tilted rectangles, whereas roles, quality, and emotional goals are attached to the appropriate functional goals and are represented by stick man icons, clouds, and heart symbols, respectively (Figure 1). Therefore, it is important that roles, quality, and emotional goals attached to the given functional goal apply to that goal and to all of the functional goals located below it in the goal tree. The described method enables the representation of functional, quality (nonfunctional), and emotional requirements for an STS in a holistic manner [30-32,35,36].

The initial functional, quality, and emotional goals identified in the literature are presented in Textbox 2.

The features identified from the literature were combined into a hierarchical goal model, with *move* as the highest-level goal—purpose of the ICT solution—and *report background info*, *set goals*, *monitor activity*, *receive feedback*, and *increase awareness* as first-level functional goals. These first-level functional goals were elaborated into lower-level functional goals and attached to their quality and emotional goals. The initial goal model derived from the literature review was presented to the experts, who elicited additional functional, quality, and emotional goals. In the final goal models, the functional, quality, and emotional goals identified from the literature are marked with an asterisk (*), and those elicited by the experts are marked with a number sign (#).

The goal models can be further elaborated into the format of user stories [39], which is one of the most prevalent ways of representing requirements in agile software engineering [73,74]. We made use of the following format of user stories, which has been adapted from Cohn [73]: “As a [user performing a certain role], I need [to perform action] to support [achieving a certain goal]”*.* We will introduce an example of elaborating goal models into user stories in the *Elaboration to User Stories* subsection.

**Initial goals identified from the literature.**

**Functional goals**

1. Report background information [20,40,44,59-61]
2. Set goals [21,24,42,45,52-54,60,62]
3. Monitor activity [21,24,42,45,62]
4. Receive feedback [21,24,42,45,51,54,62]
5. Participate in challenges [44,46,53,63]
6. Increase awareness [54,60,64,65]
7. Select challenge [12,47,50,66]
8. Create groups [15,48,63,67-69]
9. Invite friends [15,48,64,67-69]
10. View results [55,60]
11. View comparison with others [46,55,56,60,64]
12. Show health benefits [22,55]
13. Receive activity suggestions [22,55]
14. Receive links to materials [22,55]
15. Help to interpret the data [52,64]

**Quality goals**

1. Actively [5]
2. Regularly [5]
3. Personal [21,45,46,51,52,60]
4. Achievable [12,47,50,53,66]
5. Challenging [12,47,50,66]
6. Scientific [18,20,22,54,60]
7. Supportive [22]
8. Attractive [43,46,54,60,69]
9. Easy-to-use [43,46,54,60,69]
10. Automatic [43,46,54,60,69]

**Emotional goals**

1. Enjoyment [47,49,50,53,70-72]
2. Fun [47,49,50,66,70-72]
3. Involved [44,46]
4. Feel achievement [53,66]
5. Engaged [44,46,53,56,64]
6. Thrilled [53]
7. Be aware [18,20,22,54,60,64,65]

### Inclusion of Experts, Model Validation, and Improvement

The initial goal models were validated by the experts. Although previous studies with goal models have used workshops or focus groups to gather input from stakeholders [30,32,36], due to the COVID-19 situation, in this study, one-on-one interviews in the outdoors and web-based meetings were used instead. We consulted sports scientists and physical education experts (n=5) to identify the features of ICT-based interventions that can support the PA of adolescents. Some of the included experts had previous experience in the development and implementation of PA interventions in a school setting; therefore, they could highlight several aspects and experiences from the field. All the included experts were also parents, which enabled them to reflect the expectations of parents. The initial goal models were sent to the experts before the interviews, together with an explanation of the notations and the aim of the models. These aspects were repeated by the interviewer at the beginning of each interview. Thereafter, the interviewees’ thoughts about the goal models rooted in the literature and their possible improvements were obtained. All suggestions provided by the experts were documented by the interviewer in written form. In addition, feedback concerning the clarity, usability, and comprehensiveness of the agent-oriented goal models was provided. All interviews were conducted by the same researcher.

The final versions of the goal models resulting from both the literature review and the interviews with the experts are presented and explained in the *Results* section. Goals in goal models include markings that distinguish between the ideas originating in the literature and the ideas proposed by the experts. As both the ideas appearing in the literature and the ideas proposed by the experts reached a detailed level, the resulting goal models also cover the functionalities of ICT solutions that are usually represented as detailed requirements in the form of user stories [39]. In the goal models presented in the *Results* section, such functionalities are distinguished with a different color of rectangles denoting functional goals. For completeness, in the *Participate in Challenges* subsection, we also present user stories.

## Results

### Overview

**Figure 2:**
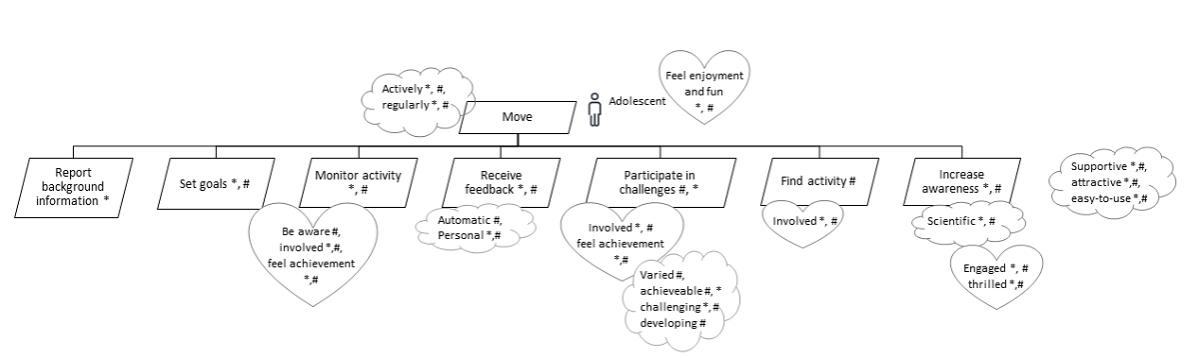
Overall goal model for information and communication technology solutions supporting physical activity levels of adolescents. The “*” in the model represents findings from the literature; the “#” represents suggestions by experts.

The outcomes of the literature review, complemented according to the opinions expressed by the experts, were represented by the goal model shown in Figure 2. The highest-level goal expressed by the goal model is that adolescents should move or, in other words, be engaged in PA. In terms of health benefits, PA should be performed actively and regularly because according to the recommendations for PA [5], adolescents should acquire every day a minimum of 60 minutes of moderate- to vigorous-intensity PA. This is reflected by the *actively* and *regularly* quality goals associated with the main goal. We also attached the emotional goals *feeling enjoyment* and *feeling fun* to the main goal, as there is an abundance of evidence that enjoyment and fun contribute to increased PA among children and youth [47,49,50,70-72]. The highest-level goal was elaborated into seven subgoals, each representing a particular aspect of achieving the highest-level goal, together with the associated quality and emotional goals.

In the following five subsections, we will explain the seven second-level functional goals represented in Figure 2 and their subgoals along with the quality and emotional goals pertaining to the functional goals.

### Report Background Information

The first activity when starting to use an ICT solution should be providing background information to offer personalized experience based on the adolescent’s personal objectives, previous PA experience, age, or gender. Using information provided by the adolescent allows the provision of messages that are more relevant to the recipient, thereby reducing information overload and supporting long-term engagement [44,59-61], while receiving personalized encouraging messages increases the effectiveness of the intervention [20,40]. A qualitative study with students confirmed that tailored information is preferred by young users [60]. Having sufficient background information can be applied for nudging [75] and directing users to healthier choices.

### Set Goals, Monitor Activity, and Receive Feedback

The inclusion of BCTs in ICT solutions for supporting PA has been emphasized by several authors, as they seem to have a significant impact on the effectiveness of the solution [15,21,22,40,41,45]. However, there is no consensus on the optimal number of BCTs that should be included in an effective ICT solution. It has been pointed out that the inclusion of multiple BCTs increases the attractiveness and effectiveness of the app [22,76]. At the same time, some reviews with adults indicate that too many BCTs can have a negative impact on effectiveness [62,77]. On average, six BCTs have been included in the apps aimed at children and adolescents [22], with the most popular BCTs being *provide instructions*, *provide general encouragement*, *provide contingent rewards* [22], *prompt specific goal setting*, *prompt self-monitoring of behavior*, and *provide feedback on performance* [21,45]. The importance of setting goals, monitoring activity, and receiving feedback was also highlighted by the experts involved in the modeling. Adding these three BCTs to the ICT-based solution can have a positive impact on the effectiveness of the solution [24,42,62]. The experts also pointed out that the inclusion of the functionalities *set goals*, *monitor activity*, and *provide feedback on performance* makes it possible to incorporate the ICT solution into teaching activities at school (eg, physical education and health education lessons), which would have multiple advantages. For example, in addition to helping students gain knowledge about their actual activity levels, incorporating the solution into in-school or in-curricula activities has the potential to increase its effectiveness [14,21] and ensure that less-motivated students also use the solution [15].

**Figure 3:**
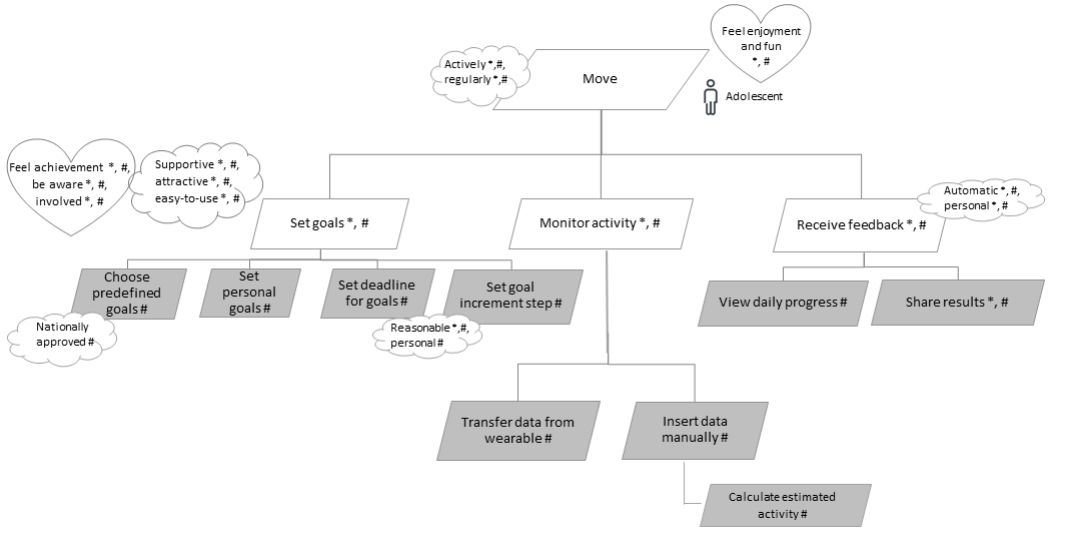
Sublevel models for the functional goals “set goals,” “monitor activity,” and “receive feedback.” The “*” in the model represents findings from the literature; the “#” represents suggestions by experts; gray rectangles represent functionalities at the level of user stories.

On the basis of the literature review and feedback from the experts, we added *set goals, monitor activity*, and *receive feedback* as the second-level functional goals. The emotional goals associated with these functional goals are *be aware, feel achievement*, and *involved*. The second-level functional goals are elaborated into sublevel functional goals, as shown in Figure 3.

For the second-level functional goal, *set goals*, shown in Figure 3, we identified that it should be possible to select from among predefined goals that are based on national and international PA recommendations as well as define one’s own goals. The benefit of defining personalized goals is that such goals match the user’s abilities and daily routines [46,51,52,60] and offer a sense of enjoyment from achieving one’s own goals [53]. Personalized goals are especially useful when goals based on national or international PA recommendations seem to be too demanding and, because of this, constantly failing to reach them would be demotivating. It is important that the goals that have been set are achievable because perceived competencies are associated with the feeling of fun [66], which, in turn, supports the continuous usage of the ICT solution and behavior change. Therefore, defining one’s goals that seem realistic and achievable supports the self-efficacy, autonomy, and motivation of adolescents. The importance of providing goal setting functionality was also highlighted by students and adolescents [52-54,60]. The experts pointed out that the possibility of setting goals enables the solution to be incorporated more easily into a school setting and use it in physical education lessons. As achievement goals should be accompanied by deadlines and sometimes it is beneficial to set subgoals with reasonable increment steps, we believe that such functionalities should be included in ICT solutions.

Goal setting functionality is strongly associated with the self-monitoring functionality to evaluate compliance with the goals that have been set. In Figure 3, this functionality is represented by the *monitor activity’s* second-level functional goal. On the basis of the literature, self-monitoring seems to be a functionality that is crucial in ICT solutions aimed at supporting PA levels [24,42,54,60,62]. Young users value self-monitoring as it helps to increase their awareness of the actual PA levels and review their progress over time [54,60,64,65], which, in turn, has been associated with increased PA levels [43,64,78]. There are many ways to measure one’s PA by, for example, using subjective ratings and questionnaires or objective data from pedometers, accelerometers, or heart rate monitors. The strength of transferring data from wearable trackers is to provide objective PA information that is more accurate compared with the information obtained by means of questionnaires and more comfortable gathering. The ICT solution should connect with wearables from different manufacturers and convert the data into an easily understandable format. However, activity trackers might underestimate some activities (eg, skiing and cycling), or it can be impossible to wear them during activities such as swimming and wrestling. In addition, users sometimes forget to wear the tracker or forget to start tracking at the beginning of the workout [55]. Therefore, it is possible to manually enter training or activity data into the ICT solution.

The third second-level functional goal shown in Figure 3—*receive feedback*—is tightly associated with goal setting and monitoring PA to evaluate performed activities and plan future ones. Providing feedback is a necessary feature pointed out by young app users [51,54], and one way to increase engagement with the solution [44] is to enhance the user’s motivation and self-efficacy [46]. Knowing that healthy PA levels have been reached increase the feeling of being healthy among adolescents [52]. However, the way feedback is given should be carefully planned and targeted so that it would not bring about negative emotions, such as guilt and disappointment [60], which can discourage and cause dropout.

Sharing results was also added to the model as one of the functional goals because sharing can increase the motivation of adolescents by receiving social support. Sharing also increases the feelings of belonging [46,56,64] and contributes positively to the effectiveness of the solution [40]. According to the experts involved in the modeling process, sharing can widen the usage of an ICT solution because it can be incorporated into physical education lessons by sharing the activity goals and results with teachers. In such a case, the physical education teacher can also serve as the provider of tailored feedback, thereby increasing the motivation of the students to be physically active. However, this possibility should be prevalidated with adolescents, as it might be perceived by them as a controlling mechanism resulting in a loss of autonomy, which can, in turn, cause resistance to using the ICT solution by adolescents [52].

### Participate in Challenges

**Figure 4:**
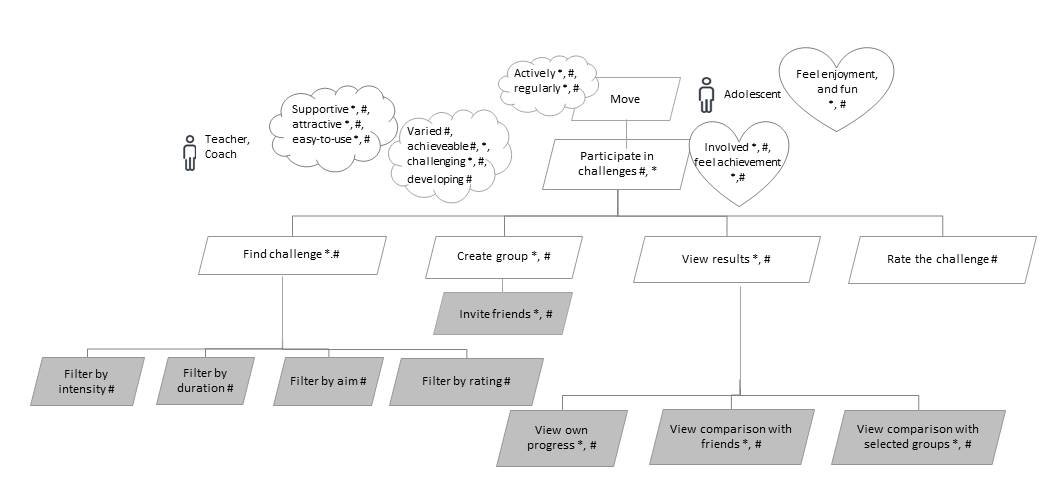
Sublevel models for the functional goal “participate in challenges.” The “*” in the model represents findings from the literature; the “#” represents suggestions by experts; gray rectangles represent functionalities at the level of user stories.

Challenges and competitions have been found to be the elements of game design that can increase engagement and reduce dropout [44,53]. In addition, participating in challenges and competitions increased the PA of the participants [46,63]. At the same time, not all people are interested in participating in challenges and competitions [12,40,46,52,53]. The studies indicate that participation in challenges is associated with PA and self-efficacy levels, as those with higher activity levels are more prone to participate in challenges [46]. It has been pointed out that competitors should have similar precompetition activity levels so that the competition effect would not drop [63] as a constant feeling of failure and no hope of winning reduces the motivation of participants [52,53]. Therefore, in addition to finding challenges, it should be possible to create groups of participants with similar PA levels or skills. This is reflected in the goal model shown in Figure 4. In addition, the ICT solution should include a variety of challenges for adolescents with different PA levels, interests, and skills, which make the participants feel competent [12,47,50,66] and would support their feeling of autonomy [50]. Moreover, the challenges have to be versatile, as similar challenges become boring for adolescents [53].

As challenges that extensively prioritize only successful performance can create subgroups of nonparticipants [56], it was suggested by the experts that the challenges should involve different kinds of targets. For example, in addition to finding the most active participants, an achievable daily threshold of a goal set by the adolescent should also be established with the aim of reaching it on all or most of the days comprised by the challenge. This approach would provide the participants with the possibility to feel a sense of achievement, increase self-efficacy, and retain anticipation to reach the goal. This also complies with the views expressed by the adolescents that not only excelling in particular sports but also merit of effort should be awarded or noticed [12]. Moreover, focusing on achieving healthy daily PA levels instead of maximum performance helps to avoid extreme PA behaviors that have been reported by some adolescents as a result of participating in PA challenges [52].

Participating in challenges is a social activity that involves interactions with other participants. The evidence suggests that participation in the challenges might be more effective when friends are engaged because there is an abundance of evidence that both the motivation to be active and actual PA levels are increased when friends are involved [15,48,67-69]. Moreover, it is more fun to be physically active together with friends [47], and peer involvement has been previously identified as a feature that adolescents like and use in ICT solutions [64]. Therefore, one should be able to invite a group of friends with whom to participate in the challenge. This is reflected by the corresponding fourth-level functional goal, *Invite friends*, shown in Figure 4. Another way of interaction during the challenge is to share information with coparticipants or a selected group of people. This is represented in Figure 4 by the third-level functional goals *view results* and *rate the challenge*. On the basis of the literature review, sharing PA information with familiar people supports PA levels, whereas sharing with strangers seems to be ineffective [46]. There are mixed feelings about comparing the results of meeting the challenge, similar to the case of participating in challenges. Some users of ICT solutions have found sharing demotivating when a comparison is made with more active people, and they rather prefer to compare themselves with lower performers to obtain assurance and confidence [55,60], whereas others find the comparison with more active people motivating [55]. According to the suggestions of the experts, we also complemented the goal model shown in Figure 4 with the roles *teacher* and *coach*, so that performers of these roles could incorporate suitable challenges into their teaching activities and would be able to view the outcomes.

### Find Activity

**Figure 5:**
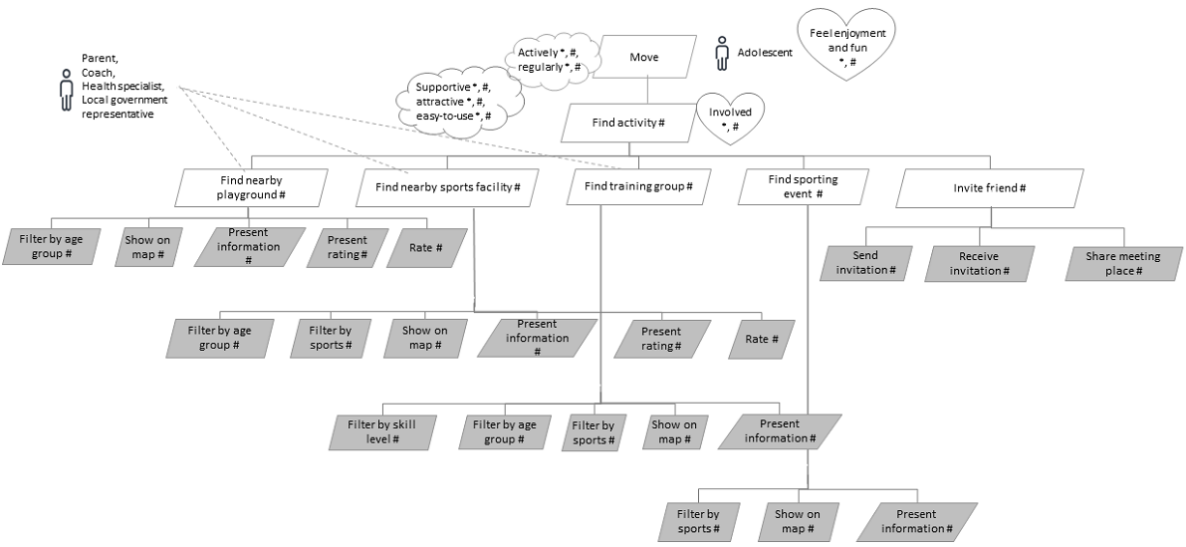
Sublevel models for functional goal “find activity.” The “*” in the model represents findings from the literature; the “#” represents suggestions by experts; gray rectangles represent functionalities at the level of user stories.

According to a socioecological model [79], one’s PA is also influenced by the built environment. To increase the awareness of PA opportunities available in the neighborhood, we have added, based on the literature, the functional goals *find nearby playground*, *find nearby sports facility, find training group*, and *find sporting event*. These functional goals along with their subgoals are presented in Figure 5. Although university students considered the feature of providing information about facilities and possibilities provided by the environment as unimportant [55], the experts involved in our study suggested that compiling a comprehensive overview of the PA opportunities in the neighborhood can support PA because free and easily accessible sports and activities are more appealing to adolescents [12]. By means of this feature, adolescents can also find places to practice nonorganized and lifestyle sports activities, such as workouts, slackline, parkour, or skateboarding, which are becoming increasingly popular among youth [80]. At the same time, this type of feature can be beneficial for different stakeholders. For example, among those who can benefit from such features are parents who want to be active with their children, teachers and coaches who want to find training places, coaches who want to be visible to their target group, and experts in public health who would like to introduce different PAs to inactive adolescents. In addition, representatives of the local government can also benefit from these features, as the ICT solution can help to identify the existing opportunities for PA and plan additional developments, considering that both proximity and accessibility of facilities affect participation in PA [47,57,66]. In addition, the presence of green spaces has the potential to increase the PA of children [81]. Therefore, the *find activity* feature would help the local government to apply nudging [75] by making healthy choices more accessible to the target group. Providing a neighborhood with opportunities for PA also reduces children’s dependence on their parents, which has been identified as one of the factors hindering adolescents from participating in sports [12].

It should be kept in mind that the preferences of adolescents vary greatly—some prefer competitive and some others prefer noncompetitive activities, and some prefer team sports and some others prefer individual sports [12,47,58]. Moreover, nonorganized and lifestyle sports activities are gaining popularity among youth [80]. To support the PA of all adolescents, different types of sports with different levels of competitiveness should be offered, as providing a choice can increase the motivation and willingness to participate [50,56]. According to the qualitative studies, adolescents value activities that are diverse, challenging, social, and support their autonomy [50] and offer the possibility to try new activities [12,82]. Adolescents should be able to distinguish between training groups based on their expected level of skills (eg, beginners, advanced, or high level) because joining a training group with more skilled participants lowers self-efficacy, reduces motivation, and increases the odds of dropout.

### Increase Awareness

**Figure 6:**
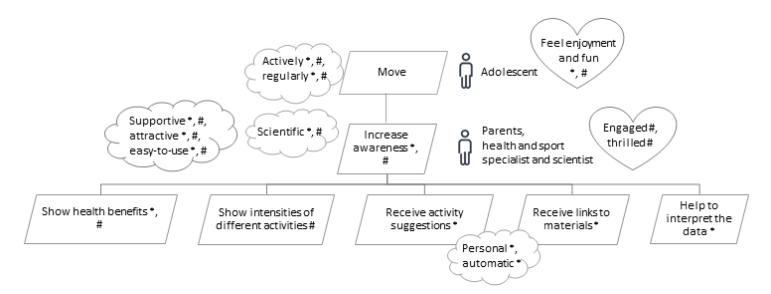
Sublevel models for the functional goal “increase awareness.” The “*” in the model represents findings from the literature; the “#” represents suggestions by experts.

Current apps aimed at youth lack health content and concrete recommendations [22,25], and only a few apps are aligned with PA recommendations [22]. The need for evidence-based content has been emphasized both by researchers [18,20,22] as well as by users of ICT solutions [54,60]. Therefore, one focus of an ICT solution should be presenting the benefits of PA. This need is reflected by the functional goal *increase awareness* and its subgoals, along with the relevant quality and emotional goals, as shown in Figure 6. This should not only include the benefits for physical health but also the benefits for social and mental health and academic achievements [1], which are less known to the wider public according to the experts who were involved in the goal modeling process of our study. In addition, developing positive attitudes toward PA and informing stakeholders on possible health gains can increase adherence to ICT solutions [24,61]. Students also reported that they would like to receive tips on how to reach their achievement goals, make activities more fun, exercise safely, and get recommendations on when it is best to exercise [55].

The feature of increasing awareness should also include providing advice on the interpretation of the collected data, as adolescents tended to neglect the information about their PA behaviors that they did not understand [52]. Providing support for data interpretation, especially at the outset of using the ICT solution, may be of critical importance to encourage adolescents to continue using the solution [64].

### Other Associated Aspects

To strengthen the effect of ICT solutions, attention should be paid to the user experience and the usefulness of the information provided, as both have been demonstrated to strengthen the effect of the solution [46,69,83]. The need for a positive user experience through a simple and well-ordered design that is pleasant to use has also been highlighted by the users involved in previous studies [43,46,54,60,69].

The opinions of adolescents concerning different gamification elements are contradictory, as some studies with adolescents have found rewarding to be an irrelevant feature [64], whereas some other studies emphasize the importance of rewards [58]. It has been suggested that different gamification elements such as avatars and rewards to achieve a goal or meet a challenge increase the attractiveness of and engagement with an ICT solution and reduce dropout [44,53]. It has also been pointed out that rewards have the potential to foster motivation and increase self-efficacy [46]. However, the logic behind receiving award points must be clear to the participants [55,69], and the reward system should be carefully planned so that it would support intrinsic motivations because intrinsically motivated behaviors are derived from enjoyment and interest by the performer [64,84]. This, in turn, supports long-term behavior changes [85]. Adolescents have pointed out that it is not just the goal achievement that should be rewarded but also the improvements and progress toward goal achievement [58].

It is widely accepted that the learning process should support the autonomy of a child, as it affects the achievement and motivation of the child [84,86]. The goal models proposed by us include elements of support for all three categories of autonomy: organizational, procedural, and cognitive. Support for organizational autonomy involves students in decision making on management issues [84], which in our goal models is expressed by the possibilities to choose group members for challenges or set dates for achievement goals. Support for procedural autonomy enables one to choose their own way of finding a solution [84], which is in our model addressed by, for example, providing information about different PA opportunities and challenges that the adolescent can choose from to achieve their goals. Support for cognitive autonomy encourages student ownership of the learning process and is considered the most important type of autonomy to be supported in the learning process [84]. Some examples of the features supporting cognitive autonomy included by our goal models are providing feedback, enabling the formulation of personal goals, and providing means to reach one’s goals independently.

### Elaboration to User Stories

In this subsection, we describe how goal models can be further elaborated to more detailed technical requirements for an ICT solution in the form of user stories [39], which is one of the most prevalent ways of representing requirements in agile software engineering [73,74]. Table 1 presents user stories associated with the submodel *Participate in challenges* shown in Figure 4, following the user story format adapted from Cohn [73], as follows: “As a [user performing a certain role], I need [to perform action] to support [achieving a certain goal]”.

**Table 1:** User stories for the submodel “Participate in challenges.”

Role and action		Goal
**Adolescent**		
	Filter by intensity	To find challenge
	Filter by duration	To find challenge
	Filter by aim	To find challenge
	Filter by rating	To find challenge
	Invite friends to participate in challenges	To create group
	View own progress	To view results
	View comparison with friends	To view results
	View comparison with selected groups	To view results
**Coach or teacher**		
	Filter by intensity	To find challenge
	Filter by duration	To find challenge
	Filter by aim	To find challenge
	Filter by rating	To find challenge
	Invite friends to participate in challenges	To create group
	View comparison with friends	To view results
	View comparison with selected groups	To view results

### Feedback From Experts

The experts considered an agent-oriented goal model to be an effective representation artifact to present the features that support the PA of adolescents and facilitate an understanding of the problem domain. The models created in our study also proved to be effective communication tools because expressing the existing scientific evidence by means of clear and simple goal models, together with just short explanations, helped the experts to understand the ideas expressed by the models and focus on analyzing and enhancing them. The created goal models provide a good overview of the necessary features of ICT solutions. The experts pointed out that although the notation of the models was easy to understand, to fully understand the presented ideas, additional information provided by the researcher was useful. It was also highlighted that the goal models created in the process could serve as input for developing ICT-based interventions because goal models presented all important and evidence-based factors in a holistic and systematic way. This would make the agent-oriented goal modeling an easily usable method for selecting and planning further intervention activities.

## Discussion

### Principal Findings

This study proposes a novel approach to create agent-oriented goal models by combining a literature review and qualitative information from experts. According to our experience, this approach is a promising and feasible method for identifying the functional, quality, and emotional goals of ICT solutions for supporting PA in adolescents. The literature review conducted by us as the first step enabled the collection of existing scientific evidence and expressed it through holistic goal models capable of representing functional, quality, and emotional aspects and relationships between them. The resulting goal models helped to obtain and present an overview of the existing problems and possible solutions and easily communicate them to nontechnical experts. As a result, the experts could focus on the aspects that were missing from the models, and they did not have to spend time or effort to point out the existing theories or previously identified possibly effective features. Such an approach also saved experts a lot of time, which is an important aspect considering the need to retain fruitful cooperation with experts and therefore cannot be underestimated. As most of the experts involved had previous experience in implementing PA interventions in a school setting or practical work experience from school, they could focus on adding valuable knowledge from the field and pointing out aspects that have a high potential to support the usability and effectiveness of ICT solutions in the target group. Examples and experiences from real life provided by experts constitute invaluable information to better understand the existing problems and possible solutions.

Several goals included by the goal models emerging from the study indicate that ICT solutions aimed at supporting PA can benefit greatly from applying machine learning methods. For example, machine learning methods can contribute to a better user experience by providing tailored advice and information. Moreover, well-timed support by the ICT solution can reduce the odds of exercise relapse or dropout [87]. In addition, machine learning algorithms can help to adjust personalized goals [87] or form adaptive goals that, according to randomized controlled trials with adults [88,89], are more effective than static goals. Therefore, future ICT developments should consider applying different machine learning methods to support the PA of users.

In this study, we preferred to conduct one-on-one interviews with experts instead of workshops or focus groups, which were often used in previous studies to identify the functional, quality, and emotional goals to be represented by goal models [30,32]. The main reason for this decision was the COVID-19 situation, which has set restrictions on physical indoor meetings in larger groups. Therefore, the interviews were conducted on the web or outdoors, where the risk of infection was lower. Such an approach certainly has its advantages and disadvantages. During one-on-one interviews, the experts were able to freely express their opinions and ideas without being interrupted, having additional discussions, or experiencing the need to further justify the ideas presented by them. In addition, the situation created by COVID-19 enabled us to refrain from *reinventing the wheel*, as the starting point of the discussions consisted of goal models created by the researcher based on a thorough literature review. On the other hand, workshops support the cocreation process in which an idea or example by one stakeholder can be developed further by the other stakeholders. The research conducted by us was a pilot study, and further studies should validate our findings and improve the goal models with a wider range of stakeholders, such as parents; coaches; and naturally, the main target group—adolescents. However, we believe that the models included in this study lay a strong foundation for further development.

### Conclusions

This paper presents the first attempt to create agent-oriented goal models of ICT solutions that support the PA of adolescents by combining evidence-based information and the opinions of experts and practitioners. The desired ICT solutions have the potential to contribute to the fight against low PA levels among adolescents by including in ICT solutions the features supported by the evidence. The strength of the proposed model is, in addition to including information from the scientific literature and experts in the field, a holistic and coherent presentation of early functional, quality, and emotional requirements. Our approach seems to be efficient in communicating the results of literature reviews to experts and supporting collaboration with nontechnical stakeholders. We believe that the created models have a high potential to help requirements engineers and developers to provide more efficient ICT solutions in the future.

## Abbreviations

BCT: behavior change technique
ICT: information and communication technology
PA: physical activity
STS: sociotechnical system

## References

[1] Janssen I, Leblanc AG (2010). Systematic review of the health benefits of physical activity and fitness in school-aged children and youth. Int J Behav Nutr Phys Act.

[2] Poitras VJ, Gray CE, Borghese MM, Carson V, Chaput J, Janssen I, Katzmarzyk PT, Pate RR, Connor Gorber S, Kho ME, Sampson M, Tremblay MS (2016). Systematic review of the relationships between objectively measured physical activity and health indicators in school-aged children and youth. Appl Physiol Nutr Metab.

[3] Strong WB, Malina RM, Blimkie CJ, Daniels SR, Dishman RK, Gutin B, Hergenroeder AC, Must A, Nixon PA, Pivarnik JM, Rowland T, Trost S, Trudeau F (2005). Evidence based physical activity for school-age youth. J Pediatr.

[4] Kalman M, Inchley J, Sigmundova D, Iannotti R, Tynjälä JA, Hamrik Z, Haug E, Bucksch J (2015). Secular trends in moderate-to-vigorous physical activity in 32 countries from 2002 to 2010: a cross-national perspective. Eur J Public Health.

[5] World Health Organization (2010). Global Recommendations on Physical Activity for Health.

[6] Dumith SC, Gigante DP, Domingues MR, Kohl HW (2011). Physical activity change during adolescence: a systematic review and a pooled analysis. Int J Epidemiol.

[7] Collings P, Wijndaele K, Corder K, Westgate K, Ridgway C, Sharp S, Dunn V, Goodyer I, Ekelund U, Brage S (2015). Magnitude and determinants of change in objectively-measured physical activity, sedentary time and sleep duration from ages 15 to 17.5y in UK adolescents: the ROOTS study. Int J Behav Nutr Phys Act.

[8] Telama R (2009). Tracking of physical activity from childhood to adulthood: a review. Obes Facts.

[9] Sawyer SM, Afifi RA, Bearinger LH, Blakemore S, Dick B, Ezeh AC, Patton GC (2012). Adolescence: a foundation for future health. Lancet.

[10] Mannocci A, D'Egidio V, Backhaus I, Federici A, Sinopoli A, Varela AR, Villari P, La Torre G (2020). Are there effective interventions to increase physical activity in children and young people? An umbrella review. Int J Environ Res Public Health.

[11] Matthews J, Win KT, Oinas-Kukkonen H, Freeman M (2016). Persuasive technology in mobile applications promoting physical activity: a systematic review. J Med Syst.

[12] Carlin A, Murphy MH, Gallagher AM (2015). Current influences and approaches to promote future physical activity in 11-13 year olds: a focus group study. BMC Public Health.

[13] Gal R, May AM, van Overmeeren EJ, Simons M, Monninkhof EM (2018). The effect of physical activity interventions comprising wearables and smartphone applications on physical activity: a systematic review and meta-analysis. Sports Med Open.

[14] Hamel LM, Robbins LB (2013). Computer- and web-based interventions to promote healthy eating among children and adolescents: a systematic review. J Adv Nurs.

[15] McIntosh JR, Jay S, Hadden N, Whittaker PJ (2017). Do e-Health interventions improve physical activity in young people: a systematic review. Public Health.

[16] Romeo A, Edney S, Plotnikoff R, Curtis R, Ryan J, Sanders I, Crozier A, Maher C (2019). Can smartphone apps increase physical activity? Systematic review and meta-analysis. J Med Internet Res.

[17] Chen J, Wilkosz ME (2014). Efficacy of technology-based interventions for obesity prevention in adolescents: a systematic review. Adolesc Health Med Ther.

[18] Rose T, Barker M, Jacob CM, Morrison L, Lawrence W, Strömmer S, Vogel C, Woods-Townsend K, Farrell D, Inskip H, Baird J (2017). A systematic review of digital interventions for improving the diet and physical activity behaviors of adolescents. J Adolesc Health.

[19] Lee AM, Chavez S, Bian J, Thompson LA, Gurka MJ, Williamson VG, Modave F (2019). Efficacy and effectiveness of mobile health technologies for facilitating physical activity in adolescents: scoping review. JMIR Mhealth Uhealth.

[20] Shin Y, Kim SK, Lee M (2019). Mobile phone interventions to improve adolescents' physical health: a systematic review and meta-analysis. Public Health Nurs.

[21] Lau PW, Lau EY, Wong DP, Ransdell L (2011). A systematic review of information and communication technology-based interventions for promoting physical activity behavior change in children and adolescents. J Med Internet Res.

[22] Schoeppe S, Alley S, Rebar AL, Hayman M, Bray NA, Van Lippevelde W, Gnam J, Bachert P, Direito A, Vandelanotte C (2017). Apps to improve diet, physical activity and sedentary behaviour in children and adolescents: a review of quality, features and behaviour change techniques. Int J Behav Nutr Phys Act.

[23] Pope L, Garnett B, Dibble M (2018). Lessons learned through the implementation of an eHealth physical activity gaming intervention with high school youth. Games Health J.

[24] Brannon EE, Cushing CC (2015). A systematic review: is there an app for that? Translational science of pediatric behavior change for physical activity and dietary interventions. J Pediatr Psychol.

[25] Schoffman DE, Turner-McGrievy G, Jones SJ, Wilcox S (2013). Mobile apps for pediatric obesity prevention and treatment, healthy eating, and physical activity promotion: just fun and games?. Transl Behav Med.

[26] Mendoza A, Miller T, Pedell S, Sterling L (2013). The role of users' emotions and associated quality goals on appropriation of systems: two case studies. Proceedings of the 24th Australasian Conference on Information Systems.

[27] Slootmaker SM, Chinapaw MJ, Seidell JC, van Mechelen W, Schuit AJ (2010). Accelerometers and internet for physical activity promotion in youth? Feasibility and effectiveness of a minimal intervention [ISRCTN93896459]. Prev Med.

[28] Curumsing MK, Fernando N, Abdelrazek M, Vasa R, Mouzakis K, Grundy J (2019). Emotion-oriented requirements engineering: a case study in developing a smart home system for the elderly. J Syst Softw.

[29] Miller T, Pedell S, Sterling L, Vetere F, Howard S (2012). Understanding socially oriented roles and goals through motivational modelling. J Syst Softw.

[30] Taveter K, Sterling L, Pedell S, Burrows R, Taveter E (2019). A method for eliciting and representing emotional requirements: two case studies in e-Healthcare. Proceedings of the IEEE 27th International Requirements Engineering Conference Workshops (REW).

[31] Sterling LS, Taveter K (2009). The Art of Agent-Oriented Modeling.

[32] Lorca A, Burrows R, Sterling L (2018). Teaching motivational models in agile requirements engineering. Proceedings of the IEEE 8th International Workshop on Requirements Engineering Education and Training (REET).

[33] Baxter G, Sommerville I (2011). Socio-technical systems: from design methods to systems engineering. Interact Comput.

[34] Sommerville I (2010). Software Engineering. 9th ed.

[35] Miller T, Lu B, Sterling L, Beydoun G, Taveter K (2014). Requirements elicitation and specification using the agent paradigm: the case study of an aircraft turnaround simulator. IIEEE Trans Software Eng.

[36] Miller T, Pedell S, Lopez-Lorca AA, Mendoza A, Sterling L, Keirnan A (2015). Emotion-led modelling for people-oriented requirements engineering: the case study of emergency systems. J Syst Softw.

[37] Shvartsman I, Taveter K, Parmak M, Meriste M (2010). Agent-oriented modelling for simulation of complex environments. Proceedings of the International Multiconference on Computer Science and Information Technology.

[38] Shvartsman I, Taveter K (2011). Agent-oriented knowledge elicitation for modeling the winning of "hearts and minds". Proceedings of the Federated Conference on Computer Science and Information Systems (FedCSIS).

[39] Tenso T, Norta A, Rootsi H, Taveter K, Vorontsova I (2017). Enhancing requirements engineering in agile methodologies by agent-oriented goal models: two empirical case studies. Proceedings of the IEEE 25th International Requirements Engineering Conference Workshops (REW).

[40] Quelly SB, Norris AE, DiPietro JL (2016). Impact of mobile apps to combat obesity in children and adolescents: a systematic literature review. J Spec Pediatr Nurs.

[41] Böhm B, Karwiese SD, Böhm H, Oberhoffer R (2019). Effects of mobile health including wearable activity trackers to increase physical activity outcomes among healthy children and adolescents: systematic review. JMIR Mhealth Uhealth.

[42] Murray JM, Brennan SF, French DP, Patterson CC, Kee F, Hunter RF (2017). Effectiveness of physical activity interventions in achieving behaviour change maintenance in young and middle aged adults: A systematic review and meta-analysis. Soc Sci Med.

[43] Ridgers ND, McNarry MA, Mackintosh KA (2016). Feasibility and effectiveness of using wearable activity trackers in youth: a systematic review. JMIR Mhealth Uhealth.

[44] Monteiro-Guerra F, Rivera-Romero O, Fernandez-Luque L, Caulfield B (2020). Personalization in real-time physical activity coaching using mobile applications: a scoping review. IEEE J Biomed Health Inform.

[45] Dute DJ, Bemelmans WJ, Breda J (2016). Using mobile apps to promote a healthy lifestyle among adolescents and students: a review of the theoretical basis and lessons learned. JMIR Mhealth Uhealth.

[46] Hosseinpour M, Terlutter R (2019). Your personal motivator is with you: a systematic review of mobile phone applications aiming at increasing physical activity. Sports Med.

[47] Casey MM, Eime RM, Payne WR, Harvey JT (2009). Using a socioecological approach to examine participation in sport and physical activity among rural adolescent girls. Qual Health Res.

[48] Jago R, Macdonald-Wallis K, Thompson JL, Page AS, Brockman R, Fox KR (2011). Better with a buddy: influence of best friends on children's physical activity. Med Sci Sports Exerc.

[49] Wilson D, Williams J, Evans A, Mixon G, Rheaume C (2005). Brief report: a qualitative study of gender preferences and motivational factors for physical activity in underserved adolescents. J Pediatr Psychol.

[50] Martins J, Marques A, Sarmento H, Carreiro da Costa F (2015). Adolescents' perspectives on the barriers and facilitators of physical activity: a systematic review of qualitative studies. Health Educ Res.

[51] Coughlin SS, Whitehead M, Sheats JQ, Mastromonico J, Smith S (2016). A review of smartphone applications for promoting physical activity. Jacobs J Community Med.

[52] Goodyear VA, Kerner C, Quennerstedt M (2017). Young people’s uses of wearable healthy lifestyle technologies; surveillance, self-surveillance and resistance. Sport Educ Soc.

[53] Corepal R, Best P, O'Neill R, Tully MA, Edwards M, Jago R, Miller SJ, Kee F, Hunter RF (2018). Exploring the use of a gamified intervention for encouraging physical activity in adolescents: a qualitative longitudinal study in Northern Ireland. BMJ Open.

[54] Dennison L, Morrison L, Conway G, Yardley L (2013). Opportunities and challenges for smartphone applications in supporting health behavior change: qualitative study. J Med Internet Res.

[55] Middelweerd A, van der Laan DM, van Stralen MM, Mollee JS, Stuij M, te Velde SJ, Brug J (2015). What features do Dutch university students prefer in a smartphone application for promotion of physical activity? A qualitative approach. Int J Behav Nutr Phys Act.

[56] Brooks F, Magnusson J (2006). Taking part counts: adolescents' experiences of the transition from inactivity to active participation in school-based physical education. Health Educ Res.

[57] Moore JB, Jilcott SB, Shores KA, Evenson KR, Brownson RC, Novick LF (2010). A qualitative examination of perceived barriers and facilitators of physical activity for urban and rural youth. Health Educ Res.

[58] Pope L, Garnett B, Dibble M (2017). Engaging adolescents to inform the development of a mobile gaming app to incentivize physical activity. JMIR Res Protoc.

[59] den Akker HO, Cabrita M, den Akker RO, Jones VM, Hermens HJ (2015). Tailored motivational message generation: a model and practical framework for real-time physical activity coaching. J Biomed Inform.

[60] Tong H, Coiera E, Laranjo L (2018). Using a mobile social networking app to promote physical activity: a qualitative study of users’ perspectives. J Med Internet Res.

[61] Yang X, Ma L, Zhao X, Kankanhalli A (2020). Factors influencing user's adherence to physical activity applications: a scoping literature review and future directions. Int J Med Inform.

[62] Michie S, Abraham C, Whittington C, McAteer J, Gupta S (2009). Effective techniques in healthy eating and physical activity interventions: a meta-regression. Health Psychol.

[63] Shameli A, Althoff T, Saberi A, Leskovec J (2017). How gamification affects physical activity: large-scale analysis of walking challenges in a mobile application. Proceedings of the 26th International Conference on World Wide Web Companion.

[64] Ridgers ND, Timperio A, Brown H, Ball K, Macfarlane S, Lai SK, Richards K, Mackintosh KA, McNarry MA, Foster M, Salmon J (2018). Wearable activity tracker use among australian adolescents: usability and acceptability study. JMIR Mhealth Uhealth.

[65] Drehlich M, Naraine M, Rowe K, Lai SK, Salmon J, Brown H, Koorts H, Macfarlane S, Ridgers ND (2020). Using the technology acceptance model to explore adolescents' perspectives on combining technologies for physical activity promotion within an intervention: usability study. J Med Internet Res.

[66] Humbert ML, Chad KE, Spink KS, Muhajarine N, Anderson KD, Bruner MW, Girolami TM, Odnokon P, Gryba CR (2006). Factors that influence physical activity participation among high- and low-SES youth. Qual Health Res.

[67] Finnerty T, Reeves S, Dabinett J, Jeanes YM, Vögele C (2009). Effects of peer influence on dietary intake and physical activity in schoolchildren. Public Health Nutr.

[68] Salvy S, Roemmich JN, Bowker JC, Romero ND, Stadler PJ, Epstein LH (2009). Effect of peers and friends on youth physical activity and motivation to be physically active. J Pediatr Psychol.

[69] Martin A, Caon M, Adorni F, Andreoni G, Ascolese A, Atkinson S, Bul K, Carrion C, Castell C, Ciociola V, Condon L, Espallargues M, Hanley J, Jesuthasan N, Lafortuna CL, Lang A, Prinelli F, Puidomenech Puig E, Tabozzi SA, McKinstry B (2020). A mobile phone intervention to improve obesity-related health behaviors of adolescents across europe: iterative co-design and feasibility study. JMIR Mhealth Uhealth.

[70] Dishman RK, Motl RW, Saunders R, Felton G, Ward DS, Dowda M, Pate RR (2005). Enjoyment mediates effects of a school-based physical-activity intervention. Med Sci Sports Exerc.

[71] Dishman R, Sallis J, Orenstein D (1985). The determinants of physical activity and exercise. Public Health Rep.

[72] Jago R, Brockman R, Fox KR, Cartwright K, Page AS, Thompson JL (2009). Friendship groups and physical activity: qualitative findings on how physical activity is initiated and maintained among 10-11 year old children. Int J Behav Nutr Phys Act.

[73] Cohn M (2004). User Stories Applied: For Agile Software Development.

[74] Paetsch F, Eberlein A, Maurer F (2003). Requirements engineering and agile software development. Proceedings of the Twelfth IEEE International Workshops on Enabling Technologies: Infrastructure for Collaborative Enterprises, 2003.

[75] Vallgårda S (2012). Nudge: a new and better way to improve health?. Health Policy.

[76] Webb TL, Joseph J, Yardley L, Michie S (2010). Using the internet to promote health behavior change: a systematic review and meta-analysis of the impact of theoretical basis, use of behavior change techniques, and mode of delivery on efficacy. J Med Internet Res.

[77] van Genugten G, Dusseldorp E, Webb TL, van Empelen P (2016). Which combinations of techniques and modes of delivery in internet-based interventions effectively change health behavior? A meta-analysis. J Med Internet Res.

[78] Gaudet J, Gallant F, Bélanger M (2017). A fit of bit: minimalist intervention in adolescents based on a physical activity tracker. JMIR Mhealth Uhealth.

[79] Dahlgren G, Whitehead M (1991). Policies and Strategies to Promote Social Equity in Health.

[80] Gilchrist P, Wheaton B (2017). The social benefits of informal and lifestyle sports: a research agenda. Int J Sport Policy Polit.

[81] Sanders T, Feng X, Fahey PP, Lonsdale C, Astell-Burt T (2015). The influence of neighbourhood green space on children's physical activity and screen time: findings from the longitudinal study of Australian children. Int J Behav Nutr Phys Act.

[82] Corder K, Schiff A, Kesten JM, van Sluijs EM (2015). Development of a universal approach to increase physical activity among adolescents: the GoActive intervention. BMJ Open.

[83] Venkatesh V, Morris MG, Davis GB, Davis FD (2003). User acceptance of information technology: toward a unified view. MIS Q.

[84] Stefanou CR, Perencevich KC, DiCintio M, Turner JC (2004). Supporting autonomy in the classroom: ways teachers encourage student decision making and ownership. Educ Psychol.

[85] Ahn SJ, Johnsen K, Ball C (2019). Points-based reward systems in gamification impact children's physical activity strategies and psychological needs. Health Educ Behav.

[86] Reeve J, Halusic M (2009). How K-12 teachers can put self-determination theory principles into practice. Theory Res Educ.

[87] Zhou M, Fukuoka Y, Goldberg K, Vittinghoff E, Aswani A (2019). Applying machine learning to predict future adherence to physical activity programs. BMC Med Inform Decis Mak.

[88] Adams MA, Sallis JF, Norman GJ, Hovell MF, Hekler EB, Perata E (2013). An adaptive physical activity intervention for overweight adults: a randomized controlled trial. PLoS One.

[89] Poirier J, Bennett WL, Jerome GJ, Shah NG, Lazo M, Yeh H, Clark JM, Cobb NK (2016). Effectiveness of an activity tracker- and internet-based adaptive walking program for adults: a randomized controlled trial. J Med Internet Res.

